# Training-Free Quantum Architecture Search Under Realistic Noise via Expressibility-Guided Evolution

**DOI:** 10.3390/e28030330

**Published:** 2026-03-16

**Authors:** Seyedali Mousavi, Seyedhamidreza Mousavi, Paul Pettersson, Masoud Daneshtalab

**Affiliations:** 1 Department of Computer Science and Engineering, Mälardalen University, 72123 Västerås, Sweden; seyedhamidreza.mousavi@mdu.se (S.M.); paul.pettersson@mdu.se (P.P.); masoud.daneshtalab@mdu.se (M.D.); 2 Department of Computer Systems, Tallinn University of Technology, 19086 Tallinn, Estonia

**Keywords:** quantum architecture search, expressibility, Kullback–Leibler divergence, information-theoretic complexity, parameterized quantum circuits, evolutionary optimization, NISQ devices

## Abstract

Designing noise-robust parameterized quantum circuits (PQCs) is a central challenge in the noisy intermediate-scale quantum (NISQ) regime. Existing quantum architecture search methods rely on training large SuperCircuits and evaluating SubCircuits under noisy execution, resulting in high computational cost and architecture assessments that depend on task-specific optimization and device noise. In this work, we propose a training-free quantum architecture search framework based on information-theoretic expressibility measures rather than performance-based estimators. We empirically show that noise-free KL-divergence-based expressibility exhibits a consistent monotonic association with noisy task loss across diverse circuit architectures and realistic hardware noise models. Leveraging this relationship, we introduce an expressibility-guided evolutionary search that requires neither SuperCircuit training nor noisy execution during the search phase. Since expressibility is evaluated independently of hardware noise, the method is inherently device-agnostic, enabling architectures to be reused across multiple quantum devices without re-running the search. Experiments using IBM-derived Qiskit noise models demonstrate that the proposed approach achieves competitive performance compared to SuperCircuit-based baselines, while substantially reducing computational cost. These results establish expressibility as an effective information-theoretic surrogate for ranking PQC architectures under realistic noise.

## 1. Introduction

Parameterized quantum circuits (PQCs) form the foundation of variational quantum algorithms in the noisy intermediate-scale quantum (NISQ) regime, enabling applications such as quantum classification, regression, and variational quantum eigensolvers (VQE) [1,2,3]. When employed within a variational optimization loop, these circuits are commonly referred to as variational quantum circuits (VQCs). The performance of a VQC depends critically on its circuit architecture, including gate composition, entanglement pattern, and depth, particularly under realistic hardware noise [3,4,5]. Designing effective circuit architectures under such constraints therefore remains a fundamental challenge.

Quantum architecture search (QAS) has recently been proposed as a systematic approach to address this challenge [6]. Among existing methods, QuantumNAS [7] introduces a noise-adaptive framework that trains a large circuit, referred to as a SuperCircuit, and performs evolutionary search over its inherited architectural subsets, termed SubCircuits. Using parameter inheritance and noisy performance estimators, this strategy significantly reduces the cost of evaluating candidate architectures compared to training each SubCircuit from scratch, while explicitly accounting for hardware noise.

Despite its effectiveness, SuperCircuit-based QAS introduces several important limitations [7,8]. First, training the SuperCircuit itself becomes increasingly costly as the circuit depth and architectural design space grow, since maintaining reliable parameter inheritance across a large population of SubCircuits typically requires substantially longer training schedules. Second, parameter inheritance tightly couples architecture evaluation to task-specific optimization dynamics, making the search process sensitive to training artifacts rather than intrinsic architectural properties. Third, and critically, when noisy execution is used as the fitness signal, the architecture evaluation requires repeated noisy simulations whose cost scales strongly with the backend size and noise-model complexity. As a result, evolutionary search can become prohibitively expensive on large-scale backends and must be repeated for each target noise model or hardware configuration. Together, these limitations motivate the exploration of training-free and device-agnostic alternatives for guiding quantum architecture search.

In this work, we propose a fundamentally different approach to quantum architecture search that decouples architecture discovery from both SuperCircuit training and device-specific noise modeling. We consider expressibility, a training-free architecture-dependent measure of a circuit’s ability to explore Hilbert space as a proxy and analyze its correlation with the noisy inherited loss across a population of candidate SubCircuits.

From an information-theoretic perspective, expressibility quantifies the statistical distance between the distribution of states generated by a parameterized circuit and the Haar-uniform distribution over Hilbert space. In this sense, the architecture search can be interpreted as selecting circuit structures that maximize the distributional coverage while maintaining robustness under realistic noise. This reframes quantum architecture design as a problem of information-theoretic complexity control rather than purely performance-driven optimization.

Based on extensive empirical analysis, we demonstrate that noise-free expressibility [4] provides a more reliable ranking signal than noise-aware alternatives, including Hilbert–Schmidt and noisy Uhlmann expressibility, the latter based on mixed-state fidelity [9]. This indicates that the structural properties of circuit architectures can be used as a low-cost pre-screening criterion, substantially reducing the need for repeated noisy evaluations during search.

Leveraging this observation, we introduce an *expressibility-guided evolutionary search* framework that performs training-free and device-agnostic architecture discovery without dataset access or explicit hardware noise models during the search phase.

Unlike QuantumNAS, which performs a co-search over both circuit structure and qubit mapping [7], we focus exclusively on SubCircuit architecture search and control for mapping effects by fixing the compilation layout. This design choice allows us to isolate the impact of circuit expressibility and provides a clean foundation for future extensions to joint architecture–mapping optimization.

We evaluate the proposed method on a quantum classification task under multiple realistic noise models derived from IBM Qiskit fake backends, which emulate gate, readout, and decoherence errors observed on real devices. The results show that the expressibility-guided search consistently outperforms the random search and approaches the performance of QuantumNAS-style SubCircuit search while requiring substantially lower computational cost.

### Contributions

The main contributions of this work are as follows:

1. We formulate PQC architecture ranking as an information-theoretic selection problem by introducing KL-based noise-free expressibility (distance to a Haar reference) as a device-independent structural ranking metric.
2. We provide a systematic empirical study of expressibility variants (noise-free, Hilbert–Schmidt, and noisy Uhlmann) and show that noise-free expressibility exhibits the most consistent monotonic association with noisy task loss across candidate architectures.
3. We introduce an expressibility-guided evolutionary search procedure that performs architecture pre-screening without SuperCircuit training or noisy execution, achieving near-comparable search quality to SuperCircuit-based methods at a substantially lower computational cost.

The remainder of the paper is organized as follows. Section 2 reviews the related work on variational quantum circuits, expressibility, and quantum architecture search. Section 3 introduces quantum state fidelity and expressibility as training-free architectural descriptors for parameterized quantum circuits. Section 4 presents the proposed expressibility-guided training-free architecture search framework. The experimental setup is described in Section 5, followed by comparative search results in Section 6. Finally, Section 7 concludes the paper and outlines directions for future research.

## 2. Related Work

### 2.1. Variational Quantum Circuits and Expressibility

Variational quantum circuits (VQCs) form the foundation of variational quantum algorithms in the noisy intermediate-scale quantum (NISQ) regime [1,2,3,10,11,12]. The choice of circuit architecture has been shown to critically influence the expressibility, trainability, and robustness under realistic hardware noise [7,13]. Sim et al. [4] formalized expressibility as a statistical distance to Haar-random states, establishing a quantitative link between circuit structure and representational capacity. Subsequent works have connected expressibility to entangling power, gradient magnitudes, and barren plateau phenomena [5,14,15].

Beyond its architectural interpretation, expressibility can be viewed from an information-theoretic perspective. Since it is defined via the statistical distance between the distribution of circuit-generated states and the Haar-uniform ensemble, it is closely related to relative entropy and other divergence-based measures used in quantum information theory [16,17]. This perspective connects the circuit architecture to the distributional complexity and provides a principled foundation for employing KL-based metrics in architecture ranking.

### 2.2. Quantum Architecture Search

Quantum architecture search (QAS) aims to automate the design of PQCs by exploring discrete structural design spaces. A comprehensive overview of QAS methodologies is provided by Martyniuk et al. [6]. Early approaches employed reinforcement learning [18], adaptive ansatz growth [19], and evolutionary strategies [20,21,22,23] to optimize circuit structures for specific tasks. Other studies explore automated optimization of variational quantum circuits using AutoML techniques, where learned performance predictors guide the joint search of circuit structures and training configurations [24].

Methods based on weight sharing and SuperCircuit training have been proposed to reduce the evaluation cost. QuantumNAS [7] introduces a noise-adaptive framework that trains a large SuperCircuit and evaluates SubCircuits via inherited parameters under noisy execution, optionally co-searching qubit mappings. One-shot QAS approaches similarly amortize training cost across architectures [8]. Despite their efficiency gains, these methods remain computationally expensive and tightly couple architecture evaluation to optimization dynamics.

### 2.3. Training-Free Architecture Search

To eliminate training overhead, training-free (or proxy-based) QAS methods have recently been proposed [25,26,27]. TF-QAS [25] introduces a progressive framework combining graph-based path counting with expressibility-driven refinement, demonstrating promising results on VQE tasks. However, TF-QAS does not systematically analyze the relationship between expressibility and noisy performance, nor does it compare noise-free and noise-aware expressibility variants.

In contrast, our work provides an explicit empirical justification for using expressibility as a primary search signal under realistic noise. We demonstrate that noise-free expressibility exhibits stronger rank correlation with noisy task loss than noise-aware alternatives, and leverage this insight to guide evolutionary search without training, noisy execution, or dataset access.

### 2.4. Hardware-Aware Compilation and Noise

Hardware noise and qubit connectivity strongly influence circuit performance. Prior work on noise-adaptive compilation and mapping seeks to mitigate these effects through optimized transpilation [28,29,30]. QuantumNAS incorporates qubit mapping into the search loop [7]. In this work, we deliberately fix compilation settings to isolate architectural effects, leaving joint architecture–mapping optimization for future study.

### 2.5. Summary of Related Work

Existing quantum architecture search methods fall into three main categories. Supercircuit-based approaches evaluate candidate architectures using inherited parameters under noisy execution, providing realistic performance estimates but incurring extremely high computational cost. Training-free heuristics reduce this cost by using structural proxies, yet these proxies are typically adopted without systematic validation under realistic noise and therefore lack reliability as search criteria. Hardware-aware compilation methods instead optimize qubit mapping and transpilation, addressing implementation efficiency rather than architectural ranking itself.

Consequently, a key missing component in the literature is a structural training-free ranking signal that is both computationally inexpensive and empirically validated to correlate with noisy task performance. This gap prevents architecture discovery from scaling to large backends without repeated noisy evaluation.

In this work, we address this limitation by showing that KL-based noise-free expressibility provides a consistent monotonic ranking signal with respect to noisy task loss. This enables architecture search to be guided by a validated structural surrogate rather than performance estimation, allowing training-free and device-agnostic quantum architecture discovery.

## 3. Fidelity and Expressibility of Parameterized Quantum Circuits

In this section, we review quantum state fidelity and circuit expressibility as architectural descriptors for PQCs. Rather than treating these quantities as direct predictors of task performance, we emphasize their role as training-free ranking-based metrics that capture the intrinsic structural properties of circuit architectures. This perspective is particularly well-suited for guiding evolutionary search, where the relative ordering of candidates is more important than absolute performance estimation.

### 3.1. Quantum State Fidelity

Quantum state fidelity is a fundamental measure of similarity between two quantum states. For general quantum states represented by density matrices ρ and σ, fidelity is defined via the Uhlmann fidelity

(1) $$F\left(\rho ,\sigma \right)={\left(\mathrm{Tr},\sqrt{\sqrt{\rho }\;\sigma \;\sqrt{\rho }}\right)}^{2},$$

which is valid for both pure and mixed states. The fidelity satisfies the bounds

(2) 0≤F(ρ,σ)≤1,

with equality to 1 if and only if ρ=σ.

For an important special case, the Uhlmann fidelity admits a simpler expression. When both states are pure, ρ=|ψ〉〈ψ| and σ=|ϕ〉〈ϕ|, Equation (1) reduces to

(3) $$F\left(\rho ,\sigma \right)={\left|\langle \psi |\phi \rangle \right|}^{2}.$$

### 3.2. Expressibility of Parameterized Quantum Circuits

Consider a parameterized quantum circuit U(θ) acting on *n* qubits, which generates a family of quantum states

(4) $$|\psi (\theta )\rangle =U\left(\theta \right){|0\rangle }^{⊗n}.$$

The expressibility of a parameterized quantum circuit characterizes how richly the circuit can explore the underlying Hilbert space as its parameters θ are varied.

A common approach to quantifying the expressibility of a quantum circuit is based on the distribution of pairwise similarities between quantum states generated by randomly sampled circuit parameters. Given two independently sampled parameter vectors $\theta_{1}$ and $\theta_{2}$, a similarity measure $S(\theta_{1},\theta_{2})$, typically state fidelity, is computed between the corresponding output states. Repeating this procedure yields an empirical similarity distribution $P_{\mathrm{circuit}}$.

As a reference for maximal expressibility, we consider the distribution of state fidelities obtained when two pure quantum states are drawn independently from the Haar measure in an *n*-qubit Hilbert space of dimension $d=2^{n}$. The Haar ensemble defines the uniform measure over quantum states and serves only as a theoretical reference; importantly, the corresponding fidelity distribution admits a closed-form probability density function and therefore does not require sampling Haar unitaries in practice.

Let

(5) $$F={\left|\langle \psi |\phi \rangle \right|}^{2}$$

denote the fidelity between two Haar-random states |ψ〉 and |ϕ〉. The resulting probability density function is

(6) $$P_{\mathrm{Haar}}\left(F\right)=\left(d-1\right){\left(1-F\right)}^{d-2}.$$

This analytic distribution serves as the baseline against which the empirical fidelity distribution generated by a parameterized circuit is compared.

The expressibility of a PQC is quantified by the Kullback–Leibler (KL) divergence between the circuit-induced similarity distribution and the Haar reference:

(7) $$\mathrm{Expr}=\mathrm{KL}\;\left(P_{\mathrm{circuit}}\;∥\;P_{\mathrm{Haar}}\right).$$

**Figure 1:**
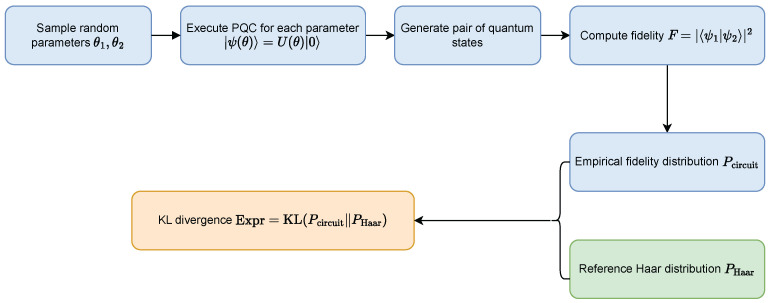
Computation of noise-free expressibility. Random circuit parameters generate quantum states whose pairwise fidelities form an empirical similarity distribution. The expressibility score is obtained by comparing this distribution with the Haar reference using KL divergence.

The practical estimation procedure used to compute expressibility is illustrated in Figure 1.

The KL divergence satisfies KL(P∥Q)≥0, with equality if and only if P=Q. Consequently, smaller values of Expr indicate that the similarity distribution induced by the circuit is closer to that of Haar-random states. Since a circuit is considered more expressive when the distribution of states it generates more closely resembles the Haar distribution, lower values of Expr correspond to higher circuit expressibility.

### 3.3. Variants of Expressibility

In practice, expressibility can be instantiated in multiple ways depending on the choice of similarity measure and noise model. In this work, we consider three variants that differ in computational cost, noise awareness, and statistical stability.

**Noise-free (pure-state) expressibility.** In the noise-free setting, circuit outputs are pure states, and similarity is measured using state fidelity as defined in Equation (3). The resulting expressibility metric depends solely on the circuit architecture and is independent of hardware noise, providing a stable and low-variance characterization of the circuit’s intrinsic expressive capacity.

**Hilbert–Schmidt (HS) expressibility.** Hilbert–Schmidt expressibility replaces fidelity with the Hilbert–Schmidt inner product between density matrices,

(8) $$S_{\mathrm{HS}}\left(\rho ,\sigma \right)=\mathrm{Tr}(\rho \;\sigma ).$$

This measure avoids matrix square roots and remains well-defined for mixed states, offering a computationally efficient and noise-aware approximation of fidelity-based expressibility.

**Noisy Uhlmann expressibility.** In the fully noise-aware setting, mixed-state similarity is quantified using the Uhlmann fidelity under a hardware noise model, as defined in Equation (1). While this variant most closely reflects physically realizable circuit behavior, it exhibits higher estimation variance and substantially increased computational overhead compared to the noise-free and Hilbert–Schmidt variants.

#### 3.3.1. Empirical Comparison

To assess the suitability of expressibility as a ranking proxy for quantum architecture search, we evaluate all three expressibility variants by measuring their Spearman rank correlation with the noisy validation loss obtained from parameter-inherited SubCircuit evaluation, which serves as a standard performance estimator in SuperCircuit-based QAS methods [7]. Specifically, for a fixed population of sampled SubCircuit architectures, we compute the noisy loss via parameter inheritance from a trained SuperCircuit under realistic hardware noise and compare the resulting ranking against rankings induced by different expressibility measures.

The resulting rank correlations are summarized in Table 1. These results indicate that noise-free expressibility provides the most reliable monotonic ranking signal with respect to the noise-aware estimator employed by QuantumNAS, despite being computed entirely without noise modeling or parameter training. In contrast, noise-aware expressibility variants exhibit substantially weaker correlation, likely due to increased estimation variance and noise-induced contraction of the effective state space.

**Figure 2:**
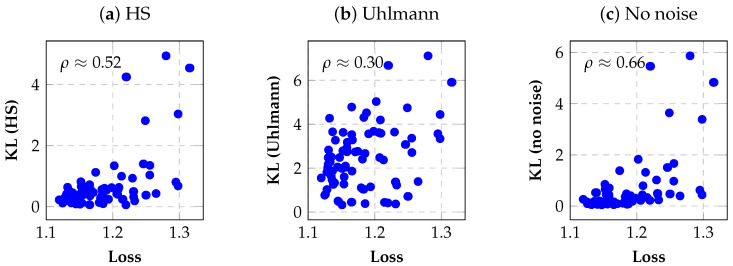
Scatter plots showing the relationship between noisy task loss and different expressibility variants across a population of candidate SubCircuits. (**a**) Hilbert–Schmidt expressibility, (**b**) noisy Uhlmann expressibility, and (**c**) noise-free expressibility. Spearman rank correlation coefficients ρ quantify the monotonic association between expressibility and noisy loss. Noise-free expressibility exhibits the strongest correlation, indicating superior ranking reliability despite being computed without hardware noise modeling.

Figure 2 visualizes these correlations by plotting noisy task loss against each expressibility variant, illustrating the superior monotonic trend induced by noise-free expressibility.

#### 3.3.2. Discussion

Although it may appear counterintuitive that noise-free expressibility correlates more strongly with noisy performance than noise-aware variants, this behavior can be explained intuitively.

First, hardware noise reduces the distinguishability between quantum states. Different circuit architectures tend to produce more similar output states once noise is applied. As a result, distribution-based metrics computed under noise exhibit higher estimation variance and reduced sensitivity to structural differences between circuits. Consequently, noisy expressibility measures may obscure meaningful architectural distinctions.

Second, expressibility characterizes the intrinsic structural capacity of a circuit, independent of how faithfully it is executed on hardware. Noise-free evaluation isolates this architectural component, providing a more stable and lower-variance ranking signal. While hardware noise influences absolute task performance, architecture determines the relative representational potential of candidate circuits, which is precisely the quantity required for an evolutionary search.

As a practical consequence, because noise-free expressibility is computed independently of any specific hardware noise model, the resulting architectural rankings are device-agnostic and can be evaluated consistently across different quantum backends without repeating the search.

Beyond explaining why noise-free expressibility correlates well with noisy performance, it is also important to consider whether the observed correlation strength is sufficient for guiding a search. Although the rank correlation (Table 1) is moderate rather than perfect, architecture search does not require accurate prediction of the absolute performance. Evolutionary optimization relies only on consistent selection pressure: higher-quality candidates should be ranked better more often than worse ones. A Spearman coefficient of ρ≈0.66 implies that, in the majority of pairwise comparisons, the proxy preserves ordering, which is sufficient to bias the search trajectory toward promising regions of the architecture space. Therefore, expressibility is not intended to replace performance evaluation as a predictor, but rather to act as a low-cost ranking heuristic that guides exploration before final training and validation.

## 4. Method: Training-Free Architecture Search via Expressibility

**Figure 3:**
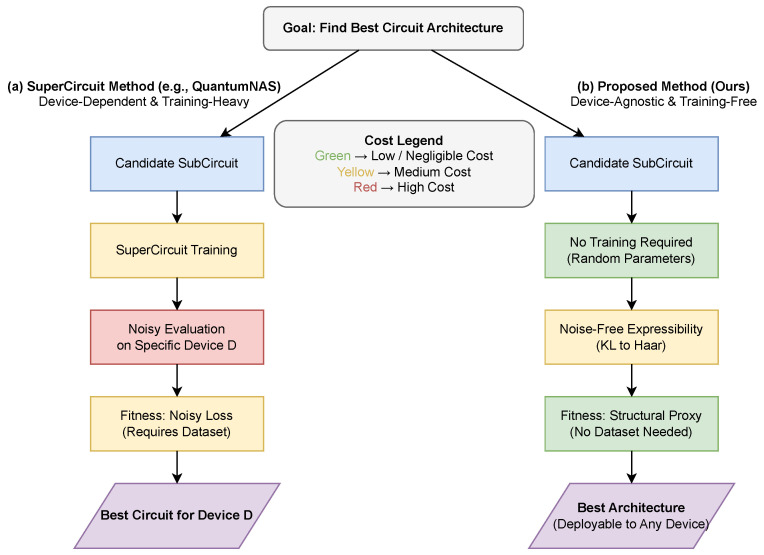
Conceptual comparison of evolutionary quantum architecture search pipelines. (**a**) SuperCircuit-based methods (e.g., QuantumNAS) rely on training a large SuperCircuit and evaluating SubCircuits via inherited parameters under noisy execution on a specific device, resulting in high training and evaluation cost and device-dependent search. (**b**) The proposed expressibility-guided approach uses a noise-free training-free structural proxy based on circuit expressibility to guide architecture ranking, reducing the need for repeated noisy performance evaluation during search and enabling device-agnostic discovery without dataset access. Colors indicate relative computational cost: red (**high**), yellow (**medium**), and green (**low**).

In this section, we introduce a training-free quantum architecture search framework that replaces performance-based estimators with expressibility-based proxies. Our method is inspired by evolutionary quantum architecture search, but it fundamentally differs from prior approaches by eliminating SuperCircuit training and parameter inheritance altogether. The overall workflow of the proposed training-free expressibility-guided architecture search is summarized in Figure 3b.

### 4.1. Problem Setting

Let A denote a PQC architecture drawn from a discrete design space S. Each architecture specifies the circuit structure, including gate types, entangling patterns, and the allocation of gates across circuit layers, while leaving the continuous variational parameters to be instantiated separately.

Given a downstream task (e.g., classification), the goal of the quantum architecture search is to identify architectures that achieve high performance under realistic noise constraints. Existing methods such as QuantumNAS estimate performance by training a SuperCircuit and evaluating SubCircuits via parameter inheritance under noisy execution. While effective, this approach introduces significant training and evaluation cost and couples architectural search to optimization dynamics.

In contrast, we seek a training-free proxy objective that depends only on the circuit structure and supports the reliable relative ranking of candidate architectures.

### 4.2. Expressibility as a Proxy Objective

Our approach is motivated by the empirical observation that expressibility exhibits a consistent monotonic association with noisy task loss across a population of architectures (Table 1). Importantly, the evolutionary architecture search depends primarily on pairwise ranking consistency rather than the accurate prediction of absolute performance. Therefore, a surrogate objective is suitable if it preserves the ordering of candidate architectures with high probability.

Based on this principle, we adopt noise-free expressibility as the fitness signal guiding the search.

For each candidate architecture A, the fitness score is defined as

(9) $$S\left(A\right)={\mathrm{Expr}}_{\mathrm{nf}}\left(A\right),$$

where ${\mathrm{Expr}}_{\mathrm{nf}}$ denotes the noise-free fidelity-based expressibility defined in Equations (3)–(7). Architectures with lower scores are considered more expressive and are preferentially selected during the evolutionary process.

Unlike noisy Uhlmann expressibility and Hilbert–Schmidt expressibility, the noise-free variant yields a low-variance architecture-dependent metric that is independent of hardware noise. As a result, expressibility needs to be computed only once per architecture and can be reused across different noise models and target devices during evaluation, rather than being re-estimated separately for each hardware configuration. This property makes noise-free expressibility particularly well suited for large-scale architecture exploration, where ranking stability, cross-device reuse, and computational efficiency are critical. Unless otherwise stated, all experiments in this work rely exclusively on ${\mathrm{Expr}}_{\mathrm{nf}}$.

#### 4.2.1. Parameter Sampling

For each architecture, circuit parameters are independently sampled from a uniform distribution

θ,ϕ,λ∼U(0,2π).

We generate $N_{\mathrm{circ}}=400$ independent parameterized circuit instances, organized into $N_{\mathrm{pairs}}=200$ disjoint pairs for fidelity computation.

This sample size represents a practical trade-off between statistical stability and computational cost. In preliminary experiments, we observed that the estimated fidelity distribution stabilizes once the number of circuit pairs exceeds approximately 150–200, with only minor changes in the resulting KL divergence values. Increasing the number of sampled pairs beyond this range provides diminishing improvements in estimation precision while proportionally increasing the simulation cost.

Since expressibility is used as a ranking signal during evolutionary search rather than as a precise estimator of absolute circuit properties, moderate Monte Carlo estimation precision is sufficient, as long as the relative ordering of candidate architectures remains stable. Empirically, we observed that the ranking induced by expressibility scores remains consistent when the number of circuit pairs varies within a reasonable range (e.g., 150–300 pairs), indicating that $N_{\mathrm{pairs}}=200$ provides a reliable and computationally efficient estimation setting.

#### 4.2.2. State Generation and Fidelity Computation

For the noise-free expressibility used during search, quantum states are simulated exactly using statevector simulation. Given two sampled parameter vectors $\theta_{1}$ and $\theta_{2}$, fidelity is computed as

$$F=|\langle \psi \left(\theta_{1}\right)|\psi \left(\theta_{2}\right)\rangle {|}^{2}.$$

No measurement sampling or shot noise is involved.

#### 4.2.3. Similarity Distribution Estimation

The empirical fidelity distribution $P_{\mathrm{circuit}}$ is estimated using a normalized histogram with 75 bins over the interval [0,1].

#### 4.2.4. Haar Reference Distribution

Instead of sampling Haar-random states, we use the analytic fidelity distribution (Equation (6)), which is integrated over histogram bins, to obtain a discrete reference distribution. This removes sampling noise from the reference distribution.

#### 4.2.5. KL Divergence Stabilization

To ensure numerical stability, both empirical and reference distributions are smoothed using additive clipping:

$$P_{i}\leftarrow \max\left(P_{i},10^{-12}\right),$$

followed by renormalization before computing the KL divergence.

### 4.3. Architecture Encoding and Design Space

Each circuit architecture is encoded as a discrete gene vector

(10) $$g=[g_{1},g_{2},\ldots ,g_{L}],$$

where each gene $g_{\ell }$ specifies the number of gates of a given type instantiated at layer *ℓ* of the circuit. The gene sequence alternates between single-qubit rotation layers and two-qubit entangling layers.

In this work, we consider a layered PQC design space composed of U3 single-qubit rotation gates and CU3 two-qubit controlled rotation gates. Odd-indexed genes correspond to U3 layers, while even-indexed genes correspond to CU3 layers. The value of each gene determines how many gates of the corresponding type are instantiated in that layer.

To illustrate this encoding, consider the example gene vector

**Figure 4:**
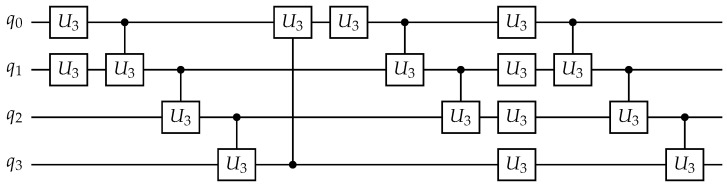
Quantum circuit instantiated from the gene vector g=[2,4,1,2,4,3] in the U3–CU3 design space with four qubits. U3 layers apply parallel single-qubit gates starting from $q_{0}$, while CU3 layers apply sequential controlled-$U_{3}$ gates following a fixed ring-based ordering $\left(q_{0}\;\to \;q_{1},\;q_{1}\;\to \;q_{2},\;q_{2}\;\to \;q_{3},\;q_{3}\;\to \;q_{0}\right)$.

(11) g=[2,4,1,2,4,3]

in a U3–CU3 design space with four qubits. This gene specifies a circuit consisting of six alternating layers: a first layer with two U3 gates, followed by a layer with four CU3 gates arranged according to a fixed ring-based qubit pairing, then one U3 gate, two CU3 gates, four U3 gates, and finally three CU3 gates. Figure 4 shows the corresponding instantiated quantum circuit generated from this gene.

During expressibility evaluation, gate parameters are sampled randomly to probe the state distribution induced by the circuit structure, while parameter optimization is performed only after the search phase on the selected architectures.

### 4.4. Expressibility-Guided Evolutionary Search

**Figure 5:**
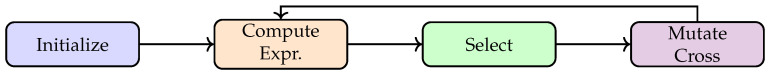
Workflow of the expressibility-guided evolutionary architecture search.

We employ an evolutionary search strategy over the discrete architecture space S. The search maintains a population of candidate architectures represented as gene vectors (Equation (11)) and iteratively refines this population through selection, mutation, and crossover. Figure 5 illustrates the workflow of this search.

#### 4.4.1. Initialization and Selection

An initial population is generated by uniformly sampling architectures from the design space S. At each generation, architectures are ranked in ascending order of their expressibility scores S(A) (Equation (9)), with lower scores indicating higher expressibility. The top-ranked candidates (i.e., those with the lowest expressibility scores) are retained as parents for the next generation.

#### 4.4.2. Mutation and Crossover

New candidate architectures are generated by applying random mutations to parent genes and by recombining gene segments from pairs of parents. Mutation probabilities, population sizes, and evolutionary hyperparameters are fixed across all compared methods to ensure a controlled comparison. The specific values are reported in Section 5.3.

#### 4.4.3. Termination

The evolutionary process is repeated for a fixed number of generations, after which the single best-ranked architecture, identified solely based on noise-free expressibility, is selected.

To account for stochasticity in both search initialization and training, the entire search-and-evaluation procedure is repeated multiple times with different random seeds, and the results are reported as the mean performance across runs.

Crucially, no parameter training, gradient computation, or dataset access is required during the search phase. The expressibility evaluation depends only on random parameter sampling and state simulation.

### 4.5. Comparison to SuperCircuit-Based Search

Compared to SuperCircuit-based approaches, our method offers several advantages:

- **No training cost**: SuperCircuit optimization is completely eliminated.
- **Architectural purity**: The evaluation depends only on circuit structure, not inherited parameters.
- **Stability**: Expressibility provides a low-variance and noise-independent ranking signal.
- **Scalability**: The computational cost scales with the number of sampled circuit instances rather than with the parameter count, training iterations, or gradient evaluations, enabling efficient exploration of larger architecture spaces.
- **Device-agnostic search**: Unlike noise-adaptive SuperCircuit-based methods, the evolutionary search is independent of hardware noise, allowing a single architecture to be discovered and reused across multiple devices without repeating the search.

These properties make expressibility-guided search particularly attractive for large or hardware-constrained architecture spaces. The complete optimization procedure is summarized in Algorithm 1.
**Algorithm 1** Expressibility-guided training-free quantum architecture search. During the search phase, architectures are ranked using noise-free expressibility evaluation only; no dataset access, gradient optimization, or noisy quantum execution is required.**Require:** Search space S, population size *P*, generations *G*, mutation probability $p_{m}$, elitism rate *e*, expressibility estimator Ω, seed *s***Ensure:** Best architecture $A^{*}$1:Initialize population $P_{0}={\left\{A_{i}\right\}}_{i=1}^{P}$ by sampling from S2:**for** each architecture $A\in P_{0}$ **do**3:   Compute expressibility ${\mathrm{Expr}}_{nf}\left(A;\Omega \right)$4:**end for**5:**for** t=0 to G−1 **do**6:   Rank $P_{t}$ by ascending ${\mathrm{Expr}}_{nf}$7:   Elitism: keep top eP architectures as $E_{t}$8:   Select parents from $E_{t}$9:   Generate offspring:
Uniform crossover between parent pairsMutation of each gene with probability $p_{m}$
10:   Form next generation $P_{t+1}=E_{t}\cup$ Offspring until size *P*11:   Compute ${\mathrm{Expr}}_{nf}$ only for new offspring (reuse cached elite scores)12:**end for**13:$A^{*}=\arg\min_{A\in P_{G}}{\mathrm{Expr}}_{nf}\left(A\right)$14:**Post-search:** Train $A^{*}$ on dataset and evaluate under noise models15:**return** $A^{*}$

## 5. Experimental Setup

This section describes the experimental configuration used to evaluate the expressibility-guided architecture search. We adopt a standardized training and evaluation protocol shared across all compared methods to ensure fair comparison.

### 5.1. Task and Dataset

We evaluate the proposed method on a four-class classification task derived from the MNIST dataset using digits {0,1,2,3}.

For the classification experiments, the dataset consists of 5000 training samples and 3000 validation samples drawn from the MNIST training set and 300 samples from the MNIST test set. The training and validation subsets are created using a 0.9/0.1 split of the available training data, while the test set is reserved for final evaluation.

### 5.2. Circuit Architecture and Design Space

Input images are first downsampled using average pooling with kernel size 6, producing a 4×4 grid of pixel intensities. These 16 values are embedded into a 4-qubit quantum circuit using a fixed angle encoding scheme.

All qubits are initialized in the state |0〉. Each row of the 4×4 feature grid is mapped to one qubit. For qubit $q_{i}$, the four pixel values in the corresponding row are interpreted as rotation angles and injected through the sequence of single-qubit rotations

$$R_{Y}\left(\theta_{i,1}\right)\to R_{Z}\left(\theta_{i,2}\right)\to R_{X}\left(\theta_{i,3}\right)\to R_{Y}\left(\theta_{i,4}\right).$$

Consequently, each qubit receives four feature-dependent rotations, resulting in a total of 16 rotations across the four-qubit register. Formally, the encoding circuit prepares the quantum state

$$|\psi \left(x\right)\rangle =U_{\mathrm{enc}}\left(x\right)\;{|0\rangle }^{⊗4},$$

where *x* denotes the classical input vector derived from the 4×4 pooled image, and $U_{\mathrm{enc}}\left(x\right)$ represents the fixed sequence of rotation gates defined above.

After encoding, the resulting quantum state is processed by the parameterized quantum circuit (PQC), which constitutes the searchable architecture in our framework. Importantly, the encoding circuit is fixed and identical for all candidate architectures, ensuring that the evolutionary search operates solely over the PQC structure. Finally, measurements in the Pauli-*Z* basis are performed on all qubits to obtain classical expectation values used for classification.

The architecture design space in this work is defined using U3 single-qubit rotations and controlled-U3 two-qubit gates (Figure 4). Although these gates are not native operations on current IBM quantum hardware, they provide a compact and hardware-agnostic parameterization of arbitrary single-qubit rotations and controlled rotations commonly used in variational quantum circuit design.

During execution on the considered IBM fake backends, circuits are automatically transpiled by Qiskit into the native basis gate set of the target device. In practice, U3 gates are decomposed into sequences of native single-qubit rotations (e.g., $R_{Z}$ and SX gates), while controlled-U3 gates are decomposed into controlled-NOT (CX) operations together with additional single-qubit rotations.

All noisy evaluations reported in this work are performed after transpilation to the backend-native gate set. Therefore, any increase in circuit depth and the associated noise overhead introduced by gate decomposition are naturally reflected in the simulated execution results. Since the same transpilation pipeline and compilation settings are applied consistently across all evaluated architectures and baselines, the comparative results remain fair despite the additional gate-level expansion introduced during compilation.

### 5.3. Evolutionary Search Hyperparameters

To ensure reproducibility, we explicitly report all evolutionary search hyperparameters used in the proposed method.

#### 5.3.1. Population and Budget

The evolutionary search maintains a fixed population of P=40 architectures per generation. Each search is executed for G=40 generations, resulting in 1600 architecture evaluations per run. All reported results are averaged over 5 independent search runs with different stochastic trajectories.

#### 5.3.2. Selection

We employ deterministic truncation selection. At each generation, architectures are ranked according to their expressibility score, and the top k=10 candidates are retained as parents. This corresponds to an elitism rate of e=25%.

#### 5.3.3. Mutation

Mutation generates 20 offspring per generation. Each gene is independently resampled from its discrete domain with probability $p_{m}=0.4$. This corresponds to categorical resampling mutation rather than local perturbation.

#### 5.3.4. Crossover

We generate 10 offspring using uniform crossover. For each gene position, the child inherits the gene from one of the two parents with equal probability 0.5.

#### 5.3.5. Constraint Handling

The search space is defined over discrete bounded gene domains. Since mutation and crossover sample only from valid gene values, all generated architectures are feasible, and no repair step is required.

#### 5.3.6. Total Search Cost

Overall, each experiment evaluates 1600 candidate architectures and 8000 architectures across all runs. Importantly, this budget is identical across all compared methods, ensuring that improvements are not due to reduced evaluation counts.

### 5.4. Training Protocol and Implementation Details

To evaluate the selected architectures, we train each candidate SubCircuit using a fixed training configuration shared across all compared methods. The optimizer is Adam with learning rate $5\times 10^{-3}$ and weight decay $10^{-4}$. Models are trained for 50 epochs with batch size 256, and the learning rate is scheduled using cosine annealing. The loss function is the negative log-likelihood (NLLLoss).

All experiments were conducted on a workstation equipped with an NVIDIA RTX A6000 GPU. Both the QuantumNAS-style baseline and the proposed training-free method were executed on this hardware configuration, and the corresponding wall-clock runtimes were measured accordingly.

### 5.5. Noise Models and Fake Backends

To simulate realistic hardware noise, we employ three IBM fake backends provided by Qiskit. Fake backends are derived from real device calibrations and are widely used as a standard benchmark for noise-aware quantum algorithm evaluation. Each fake backend represents a distinct device calibration, including gate errors, readout errors, and qubit connectivity constraints.

To evaluate the robustness across different device characteristics, we select three IBM fake backends: FakeYorktown, FakeManila, and FakeTorino. These backends are derived from calibration data of real IBM quantum devices and represent different noise profiles observed in practice.

FakeYorktown and FakeManila correspond to 5-qubit devices but originate from different hardware calibrations, resulting in distinct gate errors, readout errors, and coherence times. This allows us to evaluate whether the discovered architectures remain robust under varying calibration conditions even when the device size is fixed.

In contrast, FakeTorino represents a large-scale device derived from a 133-qubit architecture. Including this backend allows us to test the behavior of the discovered circuits under a substantially larger device topology and noise model.

While fake backends capture realistic gate, readout, and decoherence characteristics, direct execution on physical quantum hardware is left for future work as access improves.

### 5.6. Compilation and Mapping Control

All circuits are compiled using Qiskit with a fixed optimization level (level 2), which performs automatic initial qubit layout selection based on the circuit structure and the target device connectivity. To isolate the effect of the circuit architecture, we use a consistent compilation pipeline across all experiments and do not perform an explicit qubit mapping search.

By controlling the compilation process, we ensure that the observed performance differences arise from architectural variations rather than mapping artifacts or compiler-induced variability. Joint architecture–mapping optimization is left for future work.

### 5.7. Evaluation Metrics

We report the following metrics:

- **Test accuracy and test loss under noisy execution:** Performance of the trained circuits evaluated on the test set using the considered hardware noise models.
- **Mean best accuracy (μ±σ):** For each search run, the single best architecture discovered by the search procedure is trained and evaluated. We report the mean test accuracy together with the standard deviation computed across independent search runs and noise backends. This statistic summarizes both the expected performance and the stability of each search method.
- **Spearman rank correlation:** The Spearman correlation coefficient between expressibility and noisy task loss across sampled architectures, used to quantify the ranking consistency of expressibility as a proxy objective.

## 6. Search Results and Comparison

In this section, we evaluate the effectiveness of the expressibility-guided architecture search and compare it against baseline methods. Our primary goal is to assess whether expressibility alone suffices to guide the search toward high-performing SubCircuits under realistic noise conditions.

### 6.1. Baselines

We compare the proposed method against the following baselines:

- **Random Search**: Architectures are sampled uniformly at random from the design space. Each sampled SubCircuit is then trained using the same training protocol as in our method and evaluated under noisy execution. This baseline measures the benefit of guided architecture selection compared to uninformed sampling.
- **QuantumNAS-style SubCircuit Search**: An evolutionary search guided by noisy performance estimation via parameter inheritance from a trained SuperCircuit, without qubit-mapping co-search.

The goal of the comparison is not to outperform fully noise-adaptive SuperCircuit optimization but to evaluate whether architecture ranking can be approximated without training. Therefore, QuantumNAS-style search serves as an upper-bound reference for search quality under significantly higher computational cost.

### 6.2. Overall Search Performance

Table 2 summarizes the main search results across multiple noise models. For each search method, we report the test accuracy of the single best architecture identified in each run. The values are presented as the mean accuracy together with the standard deviation computed across five independent search runs and three noise backends.

Across all evaluated noise models, the expressibility-guided search consistently outperforms the random search and achieves competitive performance relative to the QuantumNAS-style SubCircuit search, despite operating without SuperCircuit training or noisy performance estimation during the search phase.

The reported standard deviations provide additional insight into the stability of the different search strategies. The random search exhibits higher variance, indicating that the quality of randomly sampled architectures varies widely. In contrast, both the QuantumNAS-style search and the proposed expressibility-guided method produce more consistent results across runs and noise backends, suggesting that these search procedures identify high-quality circuit architectures more reliably.

### 6.3. Search Efficiency

Table 3 summarizes the wall-clock time per search run. A key practical limitation of noise-aware QAS is that the search-time cost depends strongly on the target backend, because each candidate must be evaluated under a backend-specific noise model.

To make this dependency explicit, we report the QuantumNAS-style search time for FakeTorino, a 133-qubit fake backend derived from real device calibrations. Under this backend, QuantumNAS-style noisy evolutionary search required more than 4 days and 18 h to identify the best-performing SubCircuit, dominated by repeated noisy evaluations across generations.

In contrast, our expressibility-guided ranking uses noise-free simulation and depends only on the circuit structure. Consequently, the search cost is stable across backends, and the discovered architectures can be reused across devices without re-running the search.

### 6.4. Performance Across Noise Models

To assess the robustness, we evaluate each search method across three distinct IBM fake backends. Table 4 reports the mean test accuracy of the best architecture selected by each method, evaluated separately under each IBM fake backend.

The expressibility-guided search exhibits stable performance across noise models, indicating that architecture-level expressibility captures noise-robust structural properties.

### 6.5. Structural Properties of Discovered Architectures

To provide additional insight into the complexity of the circuits discovered during the search phase, we analyze the structural properties of the best-performing architectures identified by each method.

In all experiments, the number of qubits is fixed to n=4, determined by the 4×4 feature encoding described in Section 5. Consequently, differences in circuit complexity arise primarily from the number of single-qubit and two-qubit gates contained in the parameterized quantum circuits.

Each architecture is encoded as a gene vector $g=[g_{1},g_{2},\ldots ,g_{6}]$, whose elements specify the number of gates assigned to each circuit layer. Odd-indexed genes correspond to single-qubit rotation layers (U3), while even-indexed genes correspond to two-qubit entangling layers (CU3). Therefore, the number of gates in a circuit can be computed as

$$N_{U3}=g_{1}+g_{3}+g_{5},\;N_{\mathrm{CU}3}=g_{2}+g_{4}+g_{6},\;N_{\mathrm{total}}=N_{U3}+N_{\mathrm{CU}3}.$$

The statistics reported in Table 5 are computed from the final architectures obtained across five independent search runs for each method.

For the QuantumNAS-style baseline, the architecture search must be repeated for each target noise model, because candidate circuits are evaluated under device-specific noise conditions. Consequently, the reported statistics aggregate the best architectures discovered across the considered fake backends.

In contrast, the proposed expressibility-guided search is independent of hardware noise models. The architecture discovery phase is executed only once, and the resulting circuits can be evaluated on multiple quantum devices without repeating the search procedure.

These statistics reveal that the architectures discovered by the proposed expressibility-guided search tend to employ a larger number of two-qubit entangling gates compared with random search, while maintaining an overall circuit size comparable to those identified by QuantumNAS-style search. Since two-qubit gates are the primary source of entanglement in variational quantum circuits, this observation is consistent with the role of expressibility as a measure of a circuit’s ability to explore the Hilbert space. Overall, the analysis clarifies the structural complexity of the circuits identified by different search strategies and provides additional interpretability for the performance results reported earlier.

### 6.6. Summary

Overall, the results indicate that expressibility provides a consistent ranking signal for a quantum architecture search. Without SuperCircuit training or noisy performance estimation during the search phase, the proposed method outperforms the random search and approaches the performance of QuantumNAS-style SubCircuit search, while substantially reducing the computational cost.

## 7. Conclusions

We presented a training-free and device-agnostic framework for quantum architecture search based on expressibility as an information-theoretic structural proxy. By reframing architecture ranking as a distributional complexity problem, the proposed method eliminates SuperCircuit training and reduces reliance on repeated noisy performance estimation during the search phase through a KL-based measure of state-space coverage relative to a Haar reference.

Extensive empirical analysis demonstrated that noise-free expressibility exhibits a consistent monotonic association with noisy task loss across diverse architectures and realistic hardware noise models. This explains why expressibility provides a stable low-variance signal suitable for guiding evolutionary search, whereas noise-aware variants may obscure structural differences due to noise-induced manifold contraction and estimation variance.

As a result, the expressibility-guided evolutionary search achieves competitive performance relative to SuperCircuit-based methods while substantially reducing the computational cost. Because the ranking criterion is independent of hardware noise models, architectures can be discovered once and reused across multiple devices without repeating the search process.

While the observed correlation between expressibility and noisy task loss is empirically strong for the studied tasks and design spaces, it is not expected to universally predict absolute performance. Instead, the results indicate that structural capacity can guide architecture selection prior to parameter optimization, enabling efficient pre-screening of candidate circuits before expensive training.

Future work will investigate broader architecture families, larger qubit counts, and more complex variational tasks, as well as extensions toward joint architecture–mapping optimization. Overall, this work demonstrates that information-theoretic structural metrics can substantially reduce the cost of quantum architecture search while preserving most of the search quality in the NISQ regime.

## Figures and Tables

**Table 1:** Spearman rank correlation between expressibility variants and noisy task loss. Higher absolute values indicate stronger monotonic association.

Expressibility Variant	Spearman ρ
Noisy Uhlmann expressibility	≈0.30
Hilbert–Schmidt expressibility	≈0.52
**Noise-free expressibility**	≈**0.66**

**Table 2:** Comparison of architecture search methods under noisy execution. Results are reported as mean accuracy ± standard deviation computed across five independent runs and three noise backends.

Method	Mean Accuracy ± Std
Random Search	0.62±0.083
QuantumNAS-style	0.75±0.009
**Expressibility-Guided Search (Ours)**	0.71±0.019

**Table 3:** Approximate wall-clock time per search run measured on our experimental setup. For QuantumNAS-style search, the reported time includes approximately 3 h of SuperCircuit training (without noise) and 4 days 18 h of noisy evolutionary evaluation on the FakeTorino backend. For the proposed method, the search phase corresponds to noise-free expressibility evaluation. Final SubCircuit training is identical across methods.

Method	SuperCircuit Training	Search/Ranking	Final SubCircuit Training
Random Search	–	–	≈5 h
QuantumNAS-style	≈3 h	≈4 days 18 h	≈5 h
**Expressibility-Guided (Ours)**	–	≈**2 h**	≈5 h

**Table 4:** Mean test accuracy ± standard deviation across five independent runs for each IBM fake backend.

Method	FakeYorktown	FakeTorino	FakeManila
Random Search	0.61±0.053	0.61±0.094	0.63±0.101
QuantumNAS-style (SubCircuit only)	0.75±0.011	0.74±0.009	0.76±0.005
**Expressibility-Guided Search (Ours)**	0.73±0.009	0.71±0.013	0.71±0.036

**Table 5:** Structural statistics of the architectures selected by each search method before post-search training. Values show mean gate counts with the observed range across five independent runs in parentheses.

Method	Qubits	U3 Gates	CU3 Gates	Total Gates
Random Search	4	8.8 (5–11)	6.2 (5–8)	15.0 (10–18)
QuantumNAS-style	4	12.0 (10–14)	8.4 (7–9)	20.4 (17–23)
**Expressibility-Guided (Ours)**	4	12.0 (9–13)	9.4 (9–10)	21.4 (18–23)

## Data Availability

The MNIST dataset used in this study is publicly available. No new datasets were generated during this study. The implementation of the proposed framework will be made publicly available upon acceptance of the manuscript.

## References

[1] Preskill J. (2018). Quantum computing in the NISQ era and beyond. Quantum.

[2] McClean J.R., Romero J., Babbush R., Aspuru-Guzik A. (2016). The theory of variational hybrid quantum-classical algorithms. New J. Phys..

[3] Cerezo M., Arrasmith A., Babbush R., Benjamin S.C., Endo S., Fujii K., McClean J.R., Mitarai K., Yuan X., Cincio L. (2021). Variational quantum algorithms. Nat. Rev. Phys..

[4] Sim S., Johnson P.D., Aspuru-Guzik A. (2019). Expressibility and entangling capability of parameterized quantum circuits for hybrid quantum-classical algorithms. Adv. Quantum Technol..

[5] Holmes Z., Sharma K., Cerezo M., Coles P.J. (2022). Connecting ansatz expressibility to gradient magnitudes and barren plateaus. PRX Quantum.

[6] Martyniuk T., Szałkowski M., Rycerz P. (2024). Quantum architecture search: A survey. ACM Comput. Surv..

[7] Wang H., Ding Y., Tang X., Lin Y., Li Y., Fang J., Xie Y. (2022). QuantumNAS: Noise-adaptive search for robust quantum circuits. IEEE Trans. Comput.-Aided Des. Integr. Circuits Syst..

[8] Zhou Y., Tang X., Wang H., Xie Y. (2020). One-shot quantum architecture search. arXiv.

[9] Jozsa R. (1994). Fidelity for mixed quantum states. J. Mod. Opt..

[10] Benedetti M., Lloyd E., Sack S., Fiorentini M. (2019). Parameterized quantum circuits as machine learning models. Quantum Sci. Technol..

[11] Grant E., Benedetti M., Cao S., Hallam A., Lockhart J., Stojevic V., Green A.G., Severini S. (2018). Hierarchical quantum classifiers. npj Quantum Inf..

[12] Li J., Xu H., Chen G., Zhang S. (2025). Quantum Multi-View Feature Selection with Configurable Kernel Circuits and Adaptive Fusion. IEEE Trans.-Comput.-Aided Des. Integr. Circuits Syst..

[13] Nakaji K., Yamamoto N. (2021). Expressibility of the alternating layered ansatz for quantum computation. Quantum.

[14] Wang S., Fontana E., Cerezo M., Sharma K., Sone A., Cincio L., Coles P.J. (2021). Noise-induced barren plateaus in variational quantum algorithms. Nat. Commun..

[15] McClean J.R., Boixo S., Smelyanskiy V.N., Babbush R., Neven H. (2018). Barren plateaus in quantum neural network training landscapes. Nat. Commun..

[16] Vedral V. (2002). The role of relative entropy in quantum information theory. Rev. Mod. Phys..

[17] Wilde M.M. (2013). Quantum Information Theory.

[18] Zhang S.X., Chen J., Sun X. (2020). Quantum circuit architecture search with reinforcement learning. IEEE Access.

[19] Grimsley H.R., Economou S.E., Barnes E., Mayhall N.J. (2019). An adaptive variational algorithm for exact molecular simulations on a quantum computer. Nat. Commun..

[20] Huang Y., Li Q., Hou X., Wu R., Yung M.H., Bayat A., Wang X. (2022). Robust resource-efficient quantum variational ansatz through an evolutionary algorithm. Phys. Rev. A.

[21] Las Heras U., Alvarez-Rodriguez U., Solano E., Sanz M. (2016). Genetic algorithms for digital quantum simulations. Phys. Rev. Lett..

[22] Zhang A., Zhao S. (2022). Evolutionary-based quantum architecture search. arXiv.

[23] Li C., Zeng H., Ding D. (2026). Noise-Aware Quantum Architecture Search Based on NSGA-II Algorithm. arXiv.

[24] Situ H., Li Z., He Z., Li Q., Shi J. (2025). Automl-driven optimization of variational quantum circuit. Inf. Sci..

[25] He Z., Deng M., Zheng S., Li L., Situ H. Training-free quantum architecture search. Proceedings of the AAAI Conference on Artificial Intelligence.

[26] Anagolum S., Alavisamani N., Das P., Qureshi M., Shi Y. Élivágar: Efficient quantum circuit search for classification. Proceedings of the 29th ACM International Conference on Architectural Support for Programming Languages and Operating Systems.

[27] He Z., Li Z., Situ H., Li Q., Shi J., Li L. (2025). Adaptive fusion of training-free proxies for quantum architecture search. Phys. Rev. Appl..

[28] Murali P., Baker J.M., Javadi-Abhari A., Chong F.T., Martonosi M. Noise-adaptive compiler mappings for noisy intermediate-scale quantum computers. Proceedings of the 24th International Conference on Architectural Support for Programming Languages and Operating Systems (ASPLOS ’19).

[29] Li A., Ding Y., Xie Y. (2020). Pulse-efficient circuit transpilation for quantum applications. ACM International Conference on Architectural Support for Programming Languages and Operating Systems, Virtual, 16–20 March 2020.

[30] Ferrari D., Amoretti M. Noise-adaptive quantum compilation strategies evaluated with application-motivated benchmarks. Proceedings of the 19th ACM International Conference on Computing Frontiers.

